# Decoding the path to success: unveiling how behavior drives assistance dog career outcome

**DOI:** 10.3389/fvets.2026.1777631

**Published:** 2026-05-08

**Authors:** Emma Hilby, Molly McCue

**Affiliations:** Department of Population Medicine, University of Minnesota, St. Paul, MN, United States

**Keywords:** behavior assessment, behavior checklist, canine behavior, career association, service dog

## Abstract

Assistance dogs (ADs) are placed in a variety of careers to meet individual needs, mitigating disabilities, fostering independence, providing life-saving alerts, and improving mental health. Career fit can be a source of stress, particularly when an AD's placement does not align with their natural temperament. Aligning training with a dog's best-fit career may improve welfare, placement success, and overall availability of ADs for people with disabilities. Our study utilized successful (*n* = 678) assistance dogs and dogs released from training (*n* = 1,082) from six assistance dog organizations across the United States and Canada. We examined behavioral differences between ADs placed in alert (dogs who alert their partner to an impending concern their partner is unaware of, such as a drop in blood sugar, stopping with traffic coming, or a noise in the home) and response (dogs who respond to requested needs of their partners, such as retrieving dropped items or interrupting stimming behavior for children with autism) careers, as well as within specific career types. Behavior Checklist evaluations, which are the standard behavioral assessment within the AD industry, were analyzed using regression models to identify traits associated with career outcomes. For each one-unit worsening in “unwillingness to settle”, the odds of being a response dog increase by 58.7% (*p* = 0.020). For each one-unit increase in the handler-dog team, the odds of being a response dog increase by 50.2% (*p* = 0.027). Male dogs had a 60.8% increase in the odds of being a response dog, compared to female dogs (*p* = 0.004). Alert ADs were more excitable and body sensitive (not significant). Within specific careers, behavioral profiles aligned with anecdotal propensities observed by trainers. Facility dogs (dogs placed in a workplace, such as a school, hospital, or courthouse, to provide trained skills to mitigate individual stress and provide comfort) displayed behavioral differences distinct from ADs, which aligns with the difference in their career compared to assistance work. These findings demonstrate that behavioral evaluations can help predict career suitability and support informed placement decisions. By identifying both category-level and career-specific behavioral traits, AD programs can better match AD candidates to roles that align with their natural tendencies, improving both success rates and animal welfare.

## 1 Introduction

Assistance dogs (ADs, service dogs) are placed in various careers depending on the need. ADs mitigate disabilities, foster independence, provide life-saving alerts for serious medical conditions, and improve mental health by providing security and companionship. ADs are vital to the people who use them and are in high demand (1). However, organizations struggle to meet this demand—industry failure rates range from 30% to 70% due to behavioral or health issues that prevent them from being placed with a disabled partner (19). AD training organizations use a standardized behavior evaluation, the Behavior Checklist (BCL), to improve success rates. Certain behaviors, such as aggression, completely disqualify dogs from assistance work, whereas others, like body sensitivity, are career-dependent. For example, dogs with body sensitivity may still work successfully by wearing a small identification vest on their collar rather than a full vest, as is often the case for hearing alert dogs or some mobility assistance dogs. In contrast, guide dogs are required to wear a harness, and medical alert dogs must wear a pack to carry life-saving medications. Most AD organizations train for one or more careers, and successful ADs are placed in careers according to organizational needs, rather than the career in which the dog will be most successful.

Career fit may be a source of stress for ADs, especially those ADs placed in careers that conflict with their natural tendencies. The literature has thoroughly investigated the impact of ADs on their partner's stress—they significantly reduce cortisol levels, improve the partners' interpersonal interactions, and increase feelings of security for both partners and their families (1–3). However, the literature shows mixed results about how AD work impacts AD stress. Autism ADs were shown to have significant stress, likely due to the unpredictability of child behavior and routine (4). In guide ADs, cortisol levels are consistently elevated relative to those of dogs released from training (often referred to as “career change” in the AD industry), suggesting that the cognitive and emotional demands of guide work result in chronic stress (17). In contrast, PTSD ADs have lower cortisol levels than companion dogs, which might be due to the suitability of the dogs to their partners (2). AD stress is a major concern for the industry, underscoring the need for a better understanding of the behavioral characteristics best suited to specific AD careers (2, 4). However, specific behaviors that lead to success in particular AD careers are unknown. Understanding AD behavioral aptitudes has the potential to increase AD welfare by allowing organizations to: (1) evaluate puppies for desired and undesired behaviors and place them in volunteer homes that can meet the puppies' individual needs; (2) remove puppies with undesired behaviors unlikely to respond to training interventions; (3) relocate puppies to organizations where they will be better fit based on organization goals and the puppy's behavior traits; and (4) place dogs into careers that are the best fit behaviorally, improving the welfare of both the dog and the partner.

In addition to improving animal welfare, training ADs for their best-fit careers could increase success rates and ultimately increase the availability of ADs for people with disabilities. AD training programs typically report an average of 2 years to raise and train a dog from birth to partner placement. Depending on the organization, training expenses range from $30,000 to $50,000. However, this time and financial commitment do not ensure the successful completion of the program. Improving AD success by a mere 10% would considerably decrease an organization's financial burden—for example, for an organization whelping, raising, and training 100 dogs per year for $45,000 per dog, increasing the success rate by 10% would save $450,000 per year spent on unsuccessful dogs. Understanding how behavior relates to career outcomes will allow for early intervention and/or removing unsuitable dogs, shift resources toward successful placements with human partners, and increase the number of ADs available annually.

While there is some utility in identifying behaviors specific to each AD career, we believe that identifying behavioral differences between broader categories of types of assistance dogs may better help the industry in identifying dogs suited for types of assistance work. We defined these two categories, “alert” and “response”, with the following criteria: Alert dogs were classified as ADs who recognize what their partner needs before their partner does, and act accordingly. Response dogs were classified as ADs whose partner outwardly informs the dog what they need, and the dog responds to their partner's request. While there is little literature on this categorization of assistance work, we know that behavioral traits in ADs are moderately to highly heritable, and organizations purpose-breed their dogs for specific careers (5). There is evidence that hypervigilance and excitability, a trait often associated with alert dogs, have demonstrated genetic components (6, 7). While AD careers encompass a wide range of tasks, from guiding to alerting to retrieving, descriptions of these roles reveal a common underlying distinction: dogs that initiate alerts to their partners and dogs that primarily respond to human-directed requests (8). This would allow organizations to identify traits in their puppies early on and shift dogs into career categories based on attributes identified during the BCL. We believe identifying behavioral differences between alert and response ADs and placing dogs in careers based on their innate behavioral tendencies would significantly improve AD welfare.

The primary goals of this study were to better understand the differences in behavior between dogs who are successfully placed as ADs in alert and response career categories and within specific AD careers. First, we defined the behavior traits of successful ADs by categorizing careers into “response” and “alert” categories and determined the behavior differences between categories. Second, we determined the behavioral differences between specific AD careers within those broader categories. We also felt it was important to identify the common reasons for release from training in AD candidates. This included identifying the reasons for release from training for both behavioral and health reasons. Understanding behavioral fits for careers will improve career placement and allow for improved selective breeding.

## 2 Methods

### 2.1 Cohort

Members of Assistance Dogs International (ADI) use the International Working Dog Registry (IWDR) database (9, 10). There are over 100 ADI-accredited AD training organizations worldwide. These organizations use IWDR to store, standardize, and analyze data and make breeding selection and pairing decisions (5). 3,249 ADs from six partnering organizations with long-standing breeding programs and accreditation with ADI had 7,012 BCL evaluations. The distribution of careers placed at each organization is listed in Supplementary Table S3. Eight different AD careers were represented in this dataset, split into alert (guide, hearing alert, and medical alert) and response (mobility assist, autism assist, PTSD/Veteran, seizure response, and facility) categories.

### 2.2 Behavior evaluation

The BCL is a widely used behavioral assessment in the AD industry. Originally developed by several guide dog organizations and Dr. James Serpell, its application has expanded past guide dog behavior evaluation to AD organizations that train dogs for a variety of careers (5, 11). The BCL comprises 52 items, most of which are behavioral problems organizations seek to eliminate from their programs, such as body sensitivity, dog distraction, or activated/inhibited response to stressful stimuli. Guiding Eyes for the Blind and IWDR have used the BCL to generate estimated breeding values (EBVs) for breeding candidate selection; however, its utility in determining dog career outcome has not been explored (5, 12).

All participating organizations evaluated their dogs using the BCL at one to three different time points: Puppy Test (PT), Walk and Talk, and In for Final Training (IFT; see Supplementary Table S1 for details). The BCL was developed to quantify AD behavior and evaluate dog stress response with various items, including unexpected noises, objects, people, and animals (10). The BCL's temporal consistency and face validity have been evaluated (13). Each organization's scorers have been evaluated for accuracy using the International Association of Animal Behavior Consultants' Behavior Checklist certification process. Certification is valid for 2 years, with re-certification required after 2 years. Further details on the BCL, its scoring, and its use by participating organizations are available in Supplementary material S1.

### 2.3 Data analysis

R v.4.3.2 was used for all data processing and statistical analyses (18). After data cleaning (see Supplementary material S2), dogs were categorized into “alert” or “response” based on these criteria: Alert dogs were defined as assistance dogs that independently recognize their partner's needs before the partner is aware and acts on them. Response dogs were defined as assistance dogs that act after their partner outwardly communicates a specific need or request. Exploratory data analysis was performed on behavioral and medical releases to determine the most common reasons for AD release.

#### 2.3.1 Model selection

A genetic algorithm variable selection was performed on all models to eliminate predictor variables using the glmulti package in R. This approach searches the model space to determine the best models, rather than exhausting all options, which is computationally challenging with 20+ variables (14).

#### 2.3.2 Logistic regression analysis

Logistic regression models were fitted in R using the glmulti package, with the “binomial” option, to identify behavioral differences between alert and response dogs. Then, the lme4 package was used to include breed and organization as random effects. Alert was recoded as 1, and response was recoded as 0. Models that used BCL evaluations from multiple time points included scores for all BCL items that passed data filtering as predictor variables in the analysis, and the following variables were used as covariates: dog sex, breed, organization, and age at BCL evaluation. Models created for specific BCL time points and scores for all BCL items that passed data filtering were included as predictor variables. The following variables were used as covariates: dog sex, breed, and organization. See Supplementary material S3 for complete model specification.

#### 2.3.3 Univariate analysis

Univariate analyses were performed using the stats package in R for the analysis of differences in career type. The model used all BCL evaluations to identify which behaviors are different across careers. This is the first step in determining whether there are specific behavioral differences across careers before analyzing them using a more complex model. See Supplementary material S3 for complete model specification.

#### 2.3.4 Multinomial logistic regression analysis

Multinomial logistic regression models were created using the glmulti and nnet packages in R for analysis within career type. Multinomial logistic regression was fit using Firth's bias-reduced likelihood to mitigate small-sample bias and issues of separation. To achieve statistical power, we evaluated all career types with all evaluations in a single model. The comparison category was the grand mean (balanced average) of the eight career types, which can be viewed as an “average assistance dog” to compare specific careers to. Because multinomial logistic regression omits one outcome equation for identifiability, coefficients for the omitted career were reconstructed from the sum-to-zero constraint. See Supplementary material S3 for complete model specification.

## 3 Results

After data filtering for dogs with a specified career outcome and excluding dogs in training, breeding dogs, and dogs with unclear outcomes, there were 678 successful ADs and 1,082 released ADs. The success rate in this cohort was 47.5%, which is in line with the previously reported industry average. Of the dogs released, 812 were released for behavioral reasons and 270 for medical reasons (see Supplementary Table S7). When categorizing dogs as response or alert, 76% were response dogs (*n* = 515) and 24% were alert dogs (*n* = 163).

### 3.1 Comparing alert and response assistance dogs

For all analyses, sex, breed, organization, and age at BCL were all included as covariates. After model selection, the “All Evaluations,” “PT,” and “IFT” models all showed significant effect of breed (All Evaluations: Variance 0.498, SD 0.706; PT: Variance 0.312, SD 0.5588; IFT: Variance 0.367, SD 0.606) and organization (All Evaluations: Variance 2.201, SD 1.484; PT: Variance 2.190, SD 1.480; IFT: Variance 1.862, SD 1.365). In particular, success depended heavily on the organization to which a dog belonged across all three models, with success rates ranging from 35.7 to 71.1% across organizations (see Supplementary Table S3). This is likely due to the differences in career placement at organizations, as some organizations only train certain types of ADs (see Supplementary Table S3).

**Figure 1:**
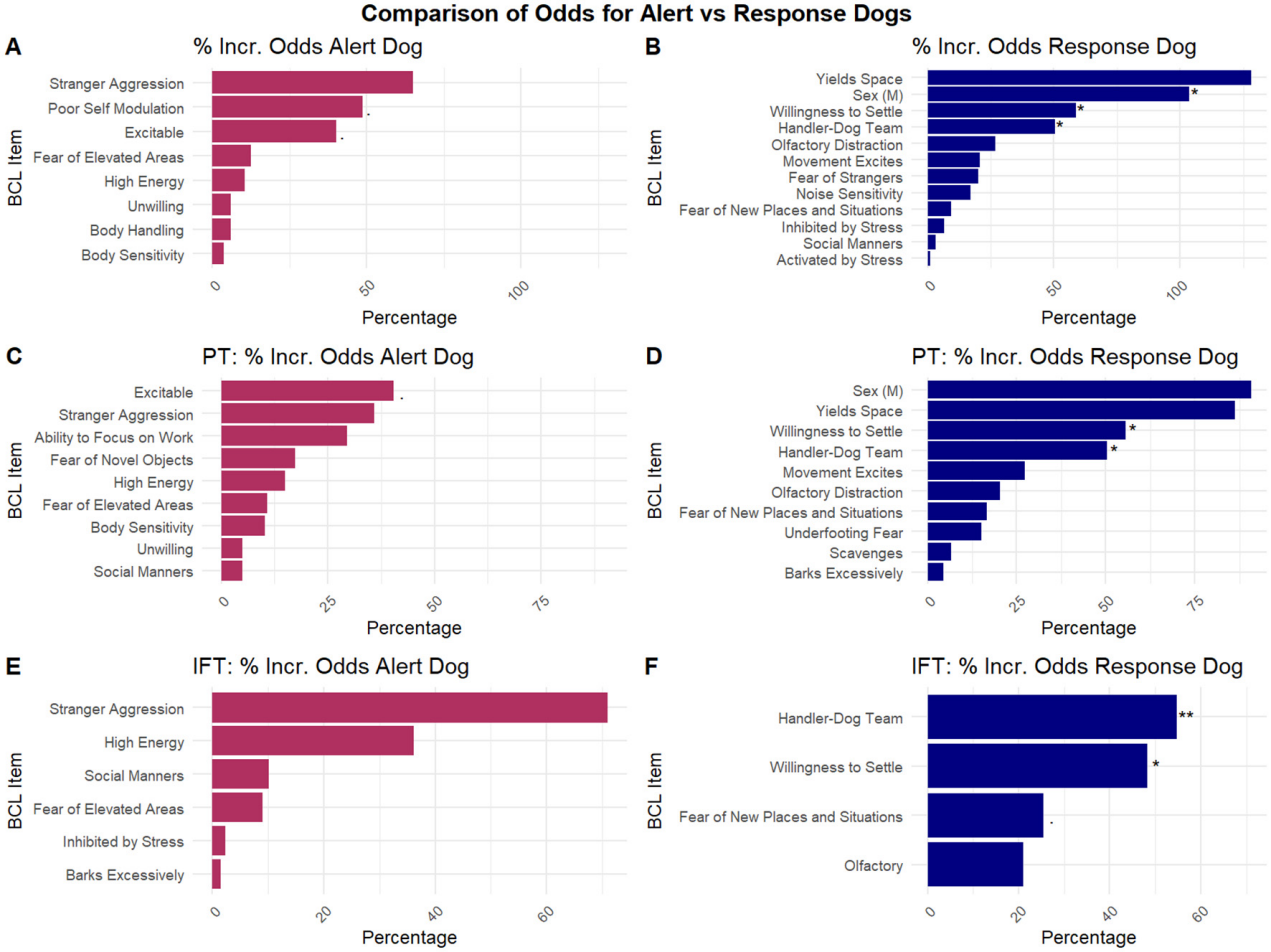
Comparison of odds between alert dogs and response dogs. *Indicates *p* < 0.05, **indicates *p* < 0.01. **(A)** Percent increase in odds for an alert dog compared to a response dog for all evaluations. **(B)** Percent increase in odds for response dog compared to alert dog for all evaluations. **(C)** Percent increase in odds for an alert dog compared to a response dog for the Puppy Test. **(D)** Percent increase in odds for response dogs compared to alert dogs for the Puppy Test. **(E)** Percent increase in odds for alert dogs compared to response dogs for the IFT. **(F)** Percent increase in odds for response dogs compared to alert dogs for the IFT.

Figures 1A, B and Supplementary Table S4 display the results for the 19 behaviors and covariates included in the “All Evaluations” model. For each one-unit worsening in “unwillingness to settle,” the odds of being a response dog increase by 58.7%. For each one-unit increase in the “handler-dog team,” the odds of being a response dog increase by 50.2%. Male dogs had a 60.8% incerase in the odds of being a response dog, compared to female dogs.

Figures 1C, D and Supplementary Table S4 display the results for 18 behaviors and covariates included in the “PT” model. For a one-unit increase in the severity of “unwillingness to settle,” the odds of being a response dog increase by 55.5%. For a one-unit increase in “handler-dog team,” the odds of being a response dog increase by 50.3%. The odds of being a response dog among male dogs increase by 90.7%.

Figures 1E, F and Supplementary Table S4 display the results for the 10 behaviors and covariates included in the “IFT” model. For a one-unit increase in the severity of “unwillingness to settle,” the odds of being a response dog increase by 48.2%. For a one-unit increase in the “handler-dog team,” the odds of being a response dog increase by 54.7%.

### 3.2 Comparing within career types

#### 3.2.1 Univariate analysis

Each behavior was used as the outcome, with career type as the single predictor. Pairwise comparisons between careers were made for each behavior. Results from this analysis are displayed in Supplementary Table S5. This investigation reveals that several careers exhibit significantly different behavioral patterns, indicating that further investigation is warranted.

#### 3.2.2 Multinomial logistic regression

For this analysis, breed, sex, organization, and age at BCL were all included as covariates. Some outcome categories and predictor combinations were sparsely represented, resulting in near-separation in the career multinomial logistic regression model. To address this, Firth's bias-reduced likelihood was used to obtain finite and stable parameter estimates. Model fit was summarized using McFadden's pseudo-*R*^2^, which was 0.64, indicating strong model fit; however, this measure does not represent explained variance and should be interpreted as a relative index of improvement over the null model.

**Figure 2:**
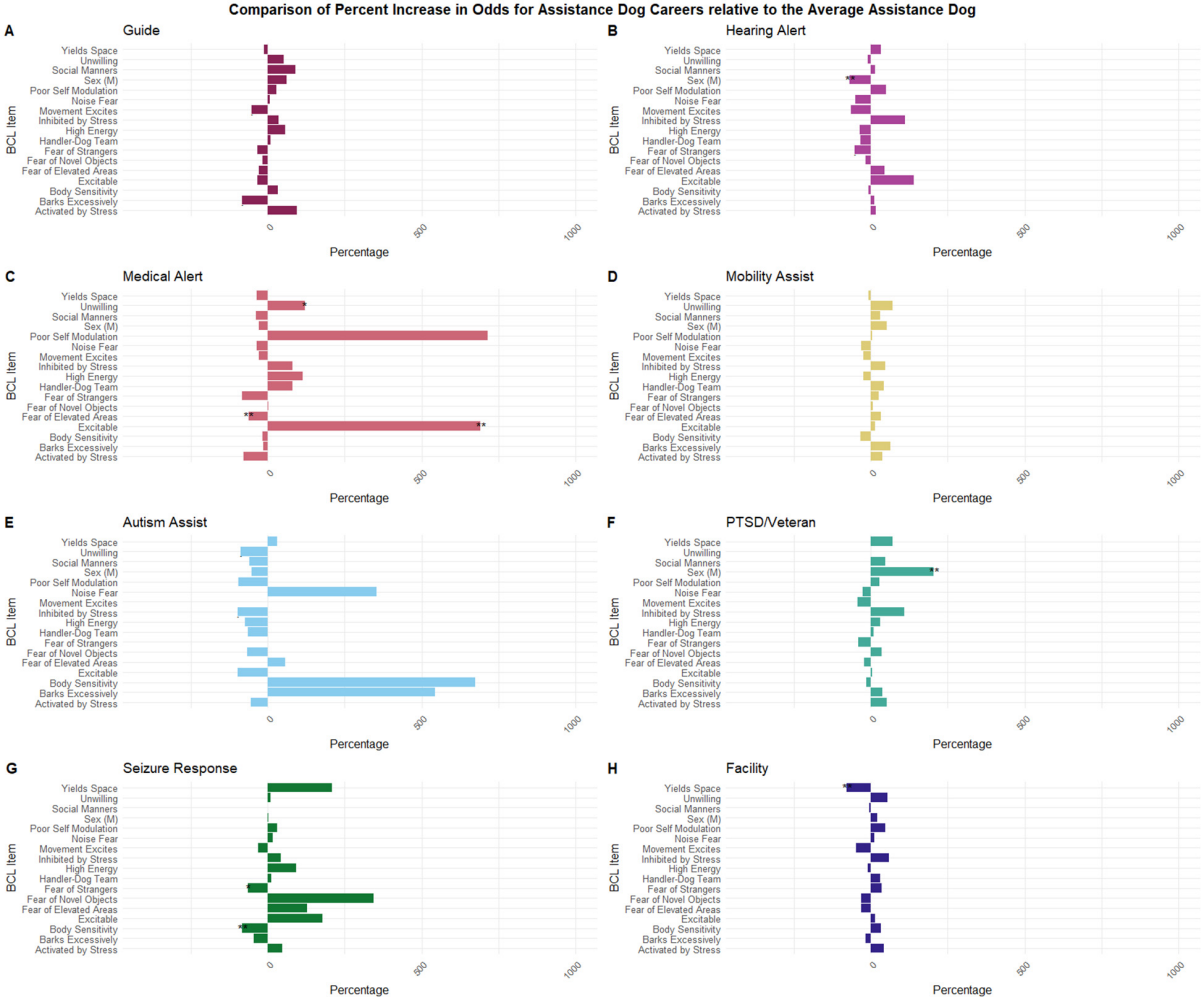
Comparison of odds between dog careers. *Indicates *p* < 0.05, **indicates *p* < 0.01. **(A)** Percent increase or decrease in odds for a guide dog compared to the grand mean. **(B)** Percent increase or decrease in odds for a hearing alert dog compared to the grand mean. **(C)** Percent increase or decrease in odds for a medical alert dog compared to the grand mean. **(D)** Percent increase or decrease in odds for a mobility assist dog compared to the grand mean. **(E)** Percent increase or decrease in odds for an autism assist dog compared to the grand mean. **(F)** Percent increase or decrease in odds for a PTSD/veteran assist compared to the grand mean. **(G)** Percent increase or decrease in odds for a seizure response dog compared to the grand mean. **(H)** Percent increase or decrease in odds for a facility dog compared to the grand mean.

For each one-unit decrease in “yields space,” the odds of being a facility dog decrease by 79% (*p* = 0.003). For each one-unit decrease in “excitability score” (dog is more excitable), the odds of being a medical alert dog increase by 692% (*p* = 0.001). For each one-unit decrease in “fear of elevated areas” (dog is more afraid), the odds of being a medical alert dog decrease by 63% (*p* = 0.004). For each one-unit decrease in “unwilling” (dog is more unwilling), the odds of being a medical alert dog increase by 120% (*p* = 0.026; meaning these dogs are more likely to pursue their own interests). For each one-unit decrease in “fear of strangers” (more afraid of strangers), the odds of being a seizure response dog decrease by 64% (*p* = 0.01) and the odds of being an autism assist dog increase by 5,209% (*p* = 0.003). For each one-unit decrease in “body sensitivity” (dog is more body sensitive), the odds of being a seizure response dog decrease by 84% (*p* = 0.001). Being a male dog increases the odds of being a veteran/PTSD dog by 205% (*p* = 0.001) and decreases the odds of being a hearing alert dog by 69% (*p* = 0.001). Figure 2 and Supplementary Table S6 display the results for all behaviors and covariates included in this analysis.

## 4 Discussion

ADs are trained to mitigate the disabilities of a variety of individuals. Although training programs now encompass multiple disability types, it remains unclear whether measurable behavioral differences distinguish ADs placed in specific careers. Most existing studies focus on single organizations and evaluate overall success or release, rather than suitability for particular AD roles. Broadly, ADs fall into two career types: response dogs, which perform tasks in response to explicit cues from their partner, and alert dogs, which independently recognize and respond to environmental or physiological cues their partner may not notice.

Anecdotally, trainers describe alert ADs as more “intense,” requiring sustained vigilance and focus, whereas response ADs are described as more “laid back,” with a greater capacity to relax between requests. While both roles demand a strong work ethic and partner responsiveness, these distinctions have not been empirically evaluated.

### 4.1 Alert and response ADs

There were more response ADs than alert ADs in the dataset, which is consistent with the current numbers reported by ADI (9). Several behaviors differentiated response and alert ADs on the PT, suggesting that meaningful behavioral distinctions can be identified early in development. This has important implications for AD organizations, particularly within ADI's International Breeding Cooperative (IBC), which routinely exchanges puppies across programs. When a litter is designated as “bred by IBC,” a portion of the puppies is distributed to other organizations, often without role-specific behavioral guidance.

While there is mixed evidence that puppy evaluations are good predictors of success in ADs, there is some utility in assessing which type of career dogs are best suited for at a young age. The IBC routinely places puppies from one organization with another organization, and knowing which behavioral propensity a puppy is likely to exhibit could assist in proper puppy placement. The logistic regression results indicate that PT evaluations show associations in early behavioral tendencies relevant to AD career type. Incorporating these findings could help organizations make more informed decisions about which puppies to retain and which to place with partnering organizations. For example, an organization that trains only response ADs and has a litter of nine puppies through IBC. Three puppies are being sent to other organizations; the organization could prioritize keeping puppies whose PT results align more closely with response work. This approach would allow organizations to retain puppies better suited to their training programs, improving the likelihood of successful placement. Similarly, labeling puppies as “alert-type” or “response-type” within IBC based on behavioral associations could help receiving organizations make informed decisions about choosing to accept or pass a puppy in their queue, reducing mismatches and increasing overall program success. There is debate in the literature on the utility of evaluating puppies: some researchers find no utility in puppy tests, while others find puppy tests are relatively predictive of later outcomes for ADs (15, 16). The analysis in this paper suggests that the PT has some associations between career placement and behavioral attributes.

The logistic regression of all behavior evaluations gave the most robust analysis of behavioral differences between alert and response ADs. Alert ADs were more excitable, had greater difficulty self-modulating, and exhibited higher energy and greater body sensitivity to both objects and handling, and, although the direction of these was not statistically significant, it is noted because this is consistent with these dogs needing to be more aware of their surroundings to perform their job. Response ADs had a higher score for handler-dog team and were far more likely to be male, which is also consistent with trainer descriptions of response dogs. Results for the PT and IFT are less robust, as there are fewer dogs in the analysis (313 BCLs in the PT analysis, 331 BCLs in IFT analysis, 678 BCLs in “All Evaluations” analysis). However, these analyses still produced behavioral differences between alert and response ADs.

While these effects were not statistically significant, several behavioral traits appeared consistently across analyses for both alert and response dogs. For alert dogs, excitability/high energy and stranger aggression were identified in all three analyses. Body handling sensitivity and sensitivity to object contact appeared in both the PT and “all” analyses. The recurrence of these traits suggests stability across age groups and indicates they may be useful associations for alert work. For response dogs, willingness to settle and fear of new places and situations, as well as handler-dog team, appeared consistently across all three analyses. This pattern suggests these traits are stable across ages and may be informative associations with response-work, particularly for dogs that show stronger handler–dog cohesion or greater difficulty adjusting to novel environments.

### 4.2 Specific careers of ADs

Determination of behavior differences between specific careers has not been investigated previously. Our results indicate that there are observable behavior differences between careers compared to the “average assistance dog” (grand mean). These results corroborate the broader analysis of AD categories, indicating that there are significant behavioral differences in AD career placement. While some behaviors were not statistically significant, many were clearly different between AD careers. Several career-behavior combinations (autism assist, seizure response, and medical alert) showed extreme point estimates with very wide CIs, suggesting quasi-separation. These estimates should be interpreted as exploratory. Hearing alert ADs were less excitable in response to movement but more excitable behaviorally, and they showed lower odds of noise fear. This pattern aligns well with their career demands: these dogs must remain highly attuned to their environment while maintaining focus on their task, often described as being constantly “on,” ready to respond to the next sound. Guide dogs had the highest odds of being activated by stress. In high-stress situations, they are still expected to perform at a high level, unlike other ADs who may be able to shut down. Activation—such as faster body movements, taking treats harder, or displaying displacement behaviors—is generally easier to manage in their career than a dog that becomes unresponsive (10). Facility dogs are very different behaviorally from all other types of ADs. Facility dogs were included in this analysis because many AD organizations also train them, and they are raised as ADs. However, they don't fit the actual definition of an assistance dog (a dog trained for one individual with a disability). This data suggests that they, perhaps, should not be included, or they should be included as a release category.

### 4.3 Releases

The most common behavioral release was environmental soundness, followed by inhibition by stress, arousal (excitable), and stranger fear and aggression. Environmental soundness encompasses noise, traffic, novel environments, objects, surfaces, and fear of riding in vehicles (see Supplementary Table S7 for details). Early environmental exposures are implemented across all AD organizations that participated in this study, yet they remain the primary reason dogs are released from their programs. This environmental enrichment for neonates before 8 weeks typically involves multiple surfaces, such as grates and uneven pavements; sound exposures, such as busy traffic noise; novel objects; and multisensory enrichment items (10). When puppies are placed in volunteer foster homes, the environmental enrichment continues with public outings and exposure to novel items in the home and on walks. This intensive intervention already suggests that organizations are attempting to address environmental soundness releases, but it remains the primary reason dogs are released from programs.

The top health release reason was for skin allergies (see Supplementary Table S7 for details). The second most common medical release was ophthalmologic eye conditions, followed by digestive issues. Skin allergies are not necessarily difficult to diagnose or treat. However, the cost associated with treating allergies is typically too much for organizations to feel comfortable imposing on a disabled individual. Most allergy medications cost more than $100 per month, which can be burdensome for individuals with existing medical expenses. It is clear from this analysis that skin allergies are a top concern of organizations, and further research is needed to assist with improving AD outcomes when allergies are suspected, or at a minimum to bring down the cost associated with allergy treatment.

### 4.4 Limitations

The BCL is a scoring system developed with a specific scale; however, the scoring of behavior itself is still subjective in nature and isn't absolute scorer to scorer. While the BCL's scorers all pass an evaluation to ensure there is some consistency in scoring, subtle differences in scorers likely remain. This study is retrospective in design, and therefore predictive capacity of behavior for career outcome cannot be determined, only association between successful outcomes and behavioral traits. It cannot be determined if behavioral profiling will actually improve placement outcomes. Organizations may release dogs early due to severe behavioral or health concerns, creating inherent survival bias in the successful cohort. Additionally, some ADs may have been placed in careers that are the best fit for an organization's needs and not the best fit for the AD. This analysis was done using six established North American ADI-accredited organizations with breeding programs and may not generalize to other types of organizations. For the multinomial logistic regression, using Firth's correction mitigates small-sample bias and separation; however, coefficient magnitudes should be interpreted in terms of direction and relative effect size rather than absolute log-odds values. In particular, estimates for organizational effects were very different, confirming that organizational structure and differences in types of dogs trained have a substantial effect and need to be accounted for.

## 5 Conclusions

Overall, our results indicate identifiable behavioral differences between response and alert ADs, as well as within individual careers, across all evaluation stages. These measurable behavior differences will give organizations the ability to make more informed decisions about ADs in their programs, whether that be that a dog's career aptitude is not a good fit for the particular organization and the AD should be transferred to better fit their personality or that a dog's career aptitude is a good fit for the organization and they can make more informed decisions about which AD candidates to keep earlier in their training careers. This analysis lays a foundation for predicting success in specific AD roles, rather than treating success as a binary category. In the future, evaluating a larger population of ADs with additional careers would strengthen the analysis and help identify clearer behavioral associations with specific successes.

## Data Availability

The datasets analyzed for this study can be provided by contacting the corresponding author.
