# Supplementary material for: Decoding the path to success: unveiling how behavior drives assistance dog career outcome
Source: Front Vet Sci. 2026 May 8;13:1777631. doi: 10.3389/fvets.2026.1777631 (PMC13193931; doi:10.3389/fvets.2026.1777631)
Supplement: Supplementary file 1 [file Data_Sheet_1.pdf]

Supplementary Material

1 Details on the Behavior Checklist

The BCL is scored on a 1 to 5 scale for most items, except two BCL items: Relationship Skills and Comparison Rating, which are scored on a 1 to 9 scale. Regardless, if a dog scores a 1 on the behavior, it is considered a severe reaction to the stimulus. If a dog scores a 5, it is considered an absent reaction to the stimuli, which is ideal. For the two items scored on a 1-9 scale, 1 is considered a poor score, and 9 is the highest possible score. Dogs are evaluated for some items multiple times throughout the BCL; for example, a dropped box of screws and a vacuum cleaner both evaluate noise sensitivity, but the dog's most severe reaction is scored. If the dog has an absent reaction to the dropped box of screws but has a mild reaction to the vacuum, the vacuum's score will be used for the dog's final BCL score.

Supplementary Table 1. Evaluation Types

**Behavior Checklist**	**Age at Evaluation**	**Description**
Puppy Test	6-8 weeks old	Puppy is brought through a series of age-appropriate novel stimuli. Novel stimuli are set up in a controlled setting, typically a room in the training facility that the puppy has never been in before. An unfamiliar handler brings the puppy through novel stimuli and the puppy's reactions are recorded.
Walk and Talk	4-10 months old	An adolescent dog is walked in a public setting (department store, home improvement store, etc.) with their puppy raiser. An instructor from the assistance dog school directs the puppy raiser through a series of stimuli and asks the raiser questions about behavioral concerns. The dog's reactions are recorded by either an independent instructor or the instructor working with the puppy raiser.
In for Final Training	15-18 months old	The dog is brought through a series of novel stimuli, which are set up in a controlled setting, typically a room in the training facility. An unfamiliar handler

Behavior Checklist	Age at Evaluation	Description
		guides the dog through the novel stimuli and the dog's reactions are recorded by independent observers from the training organization.

2 Data Cleaning

Data was first cleaned to remove dogs with non-specific careers (i.e., classified as "service dog" instead of "mobility assist") and non-specific releases (i.e., classified as "unspecified behavior release" or "unspecified medical release"). Dogs were then separated into three datasets: success, behavior release, and medical release. All cleaning and subsetting were conducted using the tidyverse package (Wickham et al. 2019).

All BCL items were recoded in reverse order to facilitate interpretation of the output. BCL items are typically scored on a one-to-five scale: one is considered a severe reaction to the stimuli, and five is considered an absent reaction. Interpreting odds ratios involves determining how the odds of the outcome change for each one-unit increase in the predictor. Because the BCL items are predictors and all BCL items are undesired behaviors, interpreting the odds ratios on the standard BCL scale was challenging; therefore, scores were reversed to improve interpretability. Additionally, data was filtered to ensure the BCL score columns had more than one unique value (i.e., ensure there isn't a column where all dogs score the same) and less than 20% missingness to remove columns with many missing values. For analyses that included multiple time points, the dog's oldest BCL evaluation was retained. Using the individual dog identification number as a random variable was considered; however, model convergence issues were suspected, and therefore, to maximize power in the analysis, each dog had only one BCL evaluation per analysis.

Supplemental Table 2. All AD types in the original dataset.

Dog Outcome	Number of Dogs	Percentage
Released	1,219	37%
In Training	832	25%
Successful ADs	785	24%
Breeding	284	9%
Transfer to Non-Participating Organization	113	3.5%
Released as Puppy	16	1.5%

Dog Outcome	Number of Dogs	Percentage
Total	3,249	100%

Supplemental Table 3. Percentage success rates for each AD organization. Numbers were calculated using only the released, successful, and breeding dogs. For this calculation, breeding dogs were considered successful. This has been anonymized.

Organization	Success Rate	Careers Placed
Organization A	71.1%	Medical Alert, Seizure Response, Mobility Assist, Autism Assist, Hearing Alert, Facility
Organization B	45.6%	Mobility Assist, Autism Assist, Veteran/PTSD, Hearing Alert, Facility
Organization C	46.7%	Veteran/PTSD
Organization D	35.7%	Mobility Assist, Veteran/PTSD, Hearing Alert, Facility
Organization E	43.9%	Seizure Response, Mobility Assist, Autism Assist, Veteran/PTSD, Hearing Alert, Facility
Organization F	41.8%	Guide, Autism Assist, Veteran/PTSD, Facility
All Organizations	47.5%	

3 Model Specification

The following equation illustrates the regression model:

$$Outcome = \mu + R1 + R2 + \dots + Rx + cov1 + \dots + covx + error$$

Where:

- Outcome is the dog's binary outcome, alert (1) or response (0)
- μ is the population average for the outcome
- Rx is each BCL item, scored from severe (recoded to 5) to absent (recoded to 1)
- Covx is the covariates included, including dog sex, breed (random effect), organization (random effect), and age at BCL evaluation
- Error represents the random error within the dataset that isn't accounted for in the model

The following equation illustrates the univariate model:

$$Behavior = \mu + Outcome + error$$

Where:

- Outcome is the dog's categorical outcome, which is either the dog's career or the dog's release reason
- μ is the population average for the behavior
- Error represents the random error within the dataset that isn't accounted for in the model

The following equation illustrates the multinomial logistic regression model:

$$Outcome = \mu + R1 + R2 + \ldots + Rx + cov1 + \ldots + covx + error$$

Where:

- Outcome is the dog's categorical outcome, which is either the dog's career or the dog's release reason
- μ is the population average for the outcome
- Rx is each BCL item, scored from severe (recoded to 5) to absent (recoded to 1)
- Covx is the covariates included, including dog sex, breed (random effect), organization (random effect), and age at BCL evaluation
- Error represents the random error within the dataset that isn't accounted for in the model

4 Comparing Alert and Response Dogs

Supplemental Table 4. Logistic regression results for All Evaluations, the Puppy Test, and the In for Final Training evaluations.

Analysis	Variable	Coefficient (β)	Standard Error	Odds Ratio (e^β)	p-value	95% CI (OR)
All Evaluations	Stranger Aggression	0.502	0.581	1.651	0.388	0.529, 5.159
	Fear of Strangers	-0.183	0.276	0.833	0.506	0.485, 1.429
	Body Handling Concern	0.057	0.157	1.058	0.718	0.778, 1.441
	Noise Fear	-0.157	0.180	0.854	0.381	0.601, 1.215
	Excitable	0.338	0.195	1.402	0.083	0.957, 2.055
	Activated by Stress	-0.010	0.171	0.990	0.951	0.707, 1.385

**Analysis**	**Variable**	**Coefficient (β)**	**Standard Error**	**Odds Ratio (e^β)**	**p-value**	**95% CI (OR)**
	Inhibited by Stress	-0.064	0.199	0.938	0.747	0.634, 1.386
	High Energy	0.099	0.247	1.104	0.688	0.680, 1.793
	Willingness to Settle	-0.462	0.199	0.630	0.020	0.426, 0.930
	Fear of Elevated Areas	0.118	0.213	1.125	0.579	0.742, 1.707
	Social Manners	-0.031	0.172	0.970	0.858	0.996, 2.218
	Poor Self Modulation	0.396	0.204	1.486	0.053	0.996, 2.217
	Olfactory Distraction	-0.239	0.171	0.787	0.163	0.563, 1.102
	Fear of New Places and Situations	-0.091	0.153	0.913	0.552	0.676, 1.232
	Yields Space	-0.825	0.512	0.438	0.107	0.161, 1.196
	Movement Excites	-0.188	0.164	0.829	0.253	0.601, 1.143
	Handler-Dog Team	-0.407	0.185	0.666	0.027	0.464, 0.956
	Unwilling	0.059	0.227	1.060	0.796	0.679, 1.656
	Body Sensitivity to Object Contact	0.036	0.156	1.036	0.820	0.763, 1.407
	Sex (M)	-0.712	0.247	0.491	0.004	0.302, 0.797
Puppy Test	Stranger Aggression	0.306	0.591	1.358	0.605	0.426, 4.332

Analysis	Variable	Coefficient (β)	Standard Error	Odds Ratio (e^β)	p-value	95% CI (OR)
	Excitable	0.340	0.195	1.405	0.081	0.959, 2.057
	Fear of Underfootings	-0.141	0.162	0.868	0.383	0.632, 1.193
	Fear of Novel Objects	0.160	0.158	1.174	0.312	0.860, 1.601
	High Energy	0.139	0.241	1.149	0.564	0.716, 1.843
	Scavenges	-0.064	0.171	0.938	0.709	0.671, 1.312
	Ability to Focus on Work	0.259	0.231	1.295	0.262	0.824, 2.035
	Willingness to Settle	-0.442	0.192	0.643	0.021	0.441, 0.937
	Fear of Elevated Areas	0.102	0.211	1.107	0.630	0.732, 1.674
	Social Manners	0.049	0.166	1.050	0.767	0.759, 1.453
	Olfactory Distraction	-0.186	0.165	0.831	0.261	0.601, 1.148
	Fear of New Places and Situations	-0.154	0.145	0.857	0.288	0.646, 1.138
	Barks Excessively	-0.043	0.218	0.958	0.843	0.625, 1.467
	Yields Space	-0.621	0.481	0.537	0.196	0.209, 1.379
	Movement Excites	-0.242	0.167	0.785	0.148	0.566, 1.089
	Handler-Dog Team	-0.407	0.183	0.665	0.026	0.465, 0.952

Analysis	Variable	Coefficient (β)	Standard Error	Odds Ratio (e^β)	p-value	95% CI (OR)
	Unwilling	0.048	0.232	1.050	0.835	0.666, 1.653
	Body Sensitivity	0.097	0.152	1.102	0.524	0.818, 1.483
	Sex (M)	-0.645	0.240	0.524	0.007	0.327, 0.840
In for Final Training	Stranger Aggression	-0.590	1.35	1.710	0.662	0.538, 5.433
	Inhibited by Stress	0.022	0.146	1.023	0.878	0.769, 1.360
	High Energy	0.308	0.201	1.361	0.125	0.918, 2.018
	Willingness to Settle	-0.394	0.176	0.675	0.025	0.478, 0.953
	Fear of Elevated Areas	0.086	0.190	1.090	0.651	0.752, 1.580
	Social Manners	0.096	0.147	1.100	0.515	0.825, 1.468
	Olfactory Distraction	-0.191	0.156	0.826	0.221	0.608, 1.122
	Fear of New Places and Situations	-0.226	0.136	0.798	0.095	0.611, 1.041
	Barks Excessively	0.015	0.202	1.015	0.940	0.683, 1.508
	Handler-Dog Team	-0.436	0.157	0.646	0.005	0.476, 0.878

4 Comparing within Careers

The most behaviorally different careers were guide dogs and mobility assist. There were nine behaviors that were significantly different between the two careers. Dogs placed as guide dogs have significantly less severe excitability, underfooting fear, and excessive barking compared to mobility

assist dogs. In contrast, dogs placed as mobility assist dogs have significantly less severe noise fear, activation with stress, inhibition with stress, scavenging, olfactory distraction, and body sensitivity. There were several careers that only had one significantly different comparison:

- Dogs placed as autism assistance dogs have significantly less severe scores for social manners than facility dog placements.
- Dogs placed as autism assistance dogs have significantly less severe scores for inhibited with stress than veteran/PTSD dogs.
- Dogs placed as facility dogs have significantly more severe scores for noise fear than medical alert dogs.
- Dogs placed as gude dogs have significantly more severe scores for body sensitivity to object contact than seizure response dogs.
- Dogs placed as seizure response dogs have significantly less severe scores for body sensitivity than veteran/PTSD dogs.

One comparison, autism assistance and medical alert, had no significant pairwise comparisons.

Supplemental Table 5. Significant pairwise comparisons between careers for each behavior. Behavior estimates are displayed in the following way: estimate (standard error); p-value.

**Career 1**	**Career 2**	**Noise Fear**	**Excitable**	**Under-footing Fear**	**Activated by Stress**	**Inhibited by Stress**	**Fear of Novel Objects**	**Scavenges**	**Ability to Focus on Work**	**Willing-ness to Settle**	**Social Manners**	**Olfact-ory**	**Barks Excess-ively**	**Body Sensitivity**
Autism	Facility										-0.458 (0.112); 0.001			
Autism	Guide				-0.415 (0.127); 0.025			-0.333 (0.107); 0.041						
Autism	Hearing		-0.449 (0.125); 0.009	-0.449 (0.125); 0.009					-0.427 (0.115); 0.005	-0.333 (0.091); 0.007	-0.477 (0.130); 0.006		-0.317 (0.087); 0.006	
Autism	Medical Alert													
Autism	Mobility		-0.396 (0.104); 0.004	-0.396 (0.104); 0.004									-0.241 (0.072); 0.019	
Autism	Veteran/PTSD					-0.343 (0.113); 0.049								
Facility	Guide				-0.406 (0.104); 0.003			-0.543 (0.088); <0.0001				-0.285 (0.092); 0.041		
Facility	Medical Alert	0.779 (0.239); 0.025												
Facility	Mobility	0.474 (0.087); <0.000				0.330 (0.083); 0.002	0.314 (0.086); 0.007				0.301 (0.082); 0.006			0.405 (0.089) <0.0001

Supplementary Material

Career 1	Career 2	Noise Fear	Excitable	Under-footing Fear	Activated by Stress	Inhibited by Stress	Fear of Novel Objects	Scavenges	Ability to Focus on Work	Willing-ness to Settle	Social Manners	Olfact-ory	Barks Excess-ively	Body Sensitivity
		1												
Facility	Seizure Response													0.873 (0.214); 0.001
Facility	Veteran/PTSD							-0.406 (0.077); <0.0001				-0.297 (0.081); 0.006		
Guide	Hearing		-0.491 (0.117); 0.001	-0.491 (0.117); 0.001	0.380 (0.125); 0.049			0.414 (0.105); 0.002	-0.410 (0.107) 0.003			0.520 (0.110); <0.0001	-0.351 (0.080); <0.0001	
Guide	Medical Alert				0.380 (0.125); 0.002			0.697 (0.198); 0.011				0.651 (0.209); 0.041		
Guide	Mobility	0.390 (0.104) ; 0.005	-0.438 (0.093); <0.0001	-0.438 (0.093); <0.0001	0.419 (0.100); 0.001	0.301 (0.098); 0.047		0.345 (0.084); 0.001				0.315 (0.088); 0.008	-0.275 (0.064); 0.001	0.326 (0.106); 0.044
Guide	Seizure Response													0.795 (0.222); 0.009
Hearing	Mobility	0.379 (0.112) ; 0.018								0.291 (0.075); 0.003	0.320 (0.105); 0.049			
Hearing	Veteran/PTSD					-0.400 (0.110); 0.007						-0.533 (0.101); <0.0001		
Medical Alert	Veteran/PTSD	-0.750 (0.239) ; 0.036			-0.720 (0.231); 0.039							-0.663 (0.205); 0.027		

Career 1	Career 2	Noise Fear	Excitable	Under-footing Fear	Activated by Stress	Inhibited by Stress	Fear of Novel Objects	Scavenges	Ability to Focus on Work	Willing-ness to Settle	Social Manners	Olfact-ory	Barks Excess-ively	Body Sensitivity
Mobility	Veteran/PTSD	-0.445 (0.086); <0.0001										-0.328 (0.076); <0.0001		-0.415 (0.091); <0.0001
Seizure Response	Veteran/PTSD													-0.884 (0.215); 0.001

Supplementary Table 6. Full results for the career comparison to the average assistance dog score for all careers, including the odds ratio and the percent increase in odds for each career and BCL item included in the model.

**Outcome**	**Predictor**	**Odds Ratio (95% CI)**	**Percentage Change in Odds**	**z Value**	**p Value**
Facility	Sex (M)	1.28 (0.49–3.34)	28%	0.5	0.617
Facility	Fear of Strangers	1.41 (0.48–4.11)	41%	0.628	0.53
Facility	Noise Fear	1.15 (0.57–2.34)	15%	0.392	0.695
Facility	Excitable	1.18 (0.52–2.68)	18%	0.406	0.684
Facility	Activated by Stress	1.50 (0.70–3.23)	50%	1.032	0.302
Facility	Inhibited by Stress	1.70 (0.74–3.89)	70%	1.246	0.213

Supplementary Material

Outcome	Predictor	Odds Ratio (95% CI)	Percentage Change in Odds	z Value	p Value
Facility	Fear of Novel Objects	0.67 (0.33–1.36)	-33%	-1.11	0.267
Facility	High Energy	0.90 (0.28–2.93)	-10%	-0.177	0.859
Facility	Fear of Elevated Areas	0.66 (0.25–1.74)	-34%	-0.841	0.4
Facility	Social Manners	0.96 (0.45–2.05)	-4%	-0.111	0.912
Facility	Poor Self Modulation	1.61 (0.64–4.07)	61%	1.017	0.309
Facility	Barks Excessively	0.87 (0.27–2.87)	-13%	-0.223	0.824
Facility	Yields Space	0.12 (0.01–1.63)	-88%	-1.59	0.112
Facility	Movement Excites	0.48 (0.24–0.95)	-52%	-2.104	0.035
Facility	Handler-Dog Team	1.37 (0.68–2.73)	37%	0.888	0.375
Facility	Unwilling	1.74 (0.61–4.96)	74%	1.037	0.3
Facility	Body Sensitivity	1.40 (0.74–2.65)	40%	1.025	0.305
Guide	Sex (M)	1.70 (0.55–5.27)	70%	0.921	0.357

Outcome	Predictor	Odds Ratio (95% CI)	Percentage Change in Odds	z Value	p Value
Guide	Fear of Strangers	0.63 (0.23–1.71)	-37%	-0.902	0.367
Guide	Noise Fear	1.08 (0.48–2.41)	8%	0.184	0.854
Guide	Excitable	0.62 (0.23–1.67)	-38%	-0.949	0.342
Guide	Activated by Stress	2.29 (0.86–6.11)	129%	1.66	0.097
Guide	Inhibited by Stress	1.41 (0.50–3.99)	41%	0.647	0.518
Guide	Fear of Novel Objects	0.86 (0.36–2.04)	-14%	-0.34	0.734
Guide	High Energy	1.55 (0.49–4.93)	55%	0.75	0.454
Guide	Fear of Elevated Areas	0.66 (0.27–1.61)	-34%	-0.916	0.359
Guide	Social Manners	2.24 (0.94–5.34)	124%	1.821	0.069
Guide	Poor Self Modulation	1.37 (0.54–3.49)	37%	0.661	0.508
Guide	Barks Excessively	0.09 (0.01–0.98)	-91%	-1.976	0.048
Guide	Yields Space	0.83 (0.13–5.27)	-17%	-0.197	0.844

Supplementary Material

Outcome	Predictor	Odds Ratio (95% CI)	Percentage Change in Odds	z Value	p Value
Guide	Movement Excites	0.41 (0.18–0.92)	-59%	-2.169	0.03
Guide	Handler-Dog Team	1.10 (0.47–2.59)	10%	0.222	0.824
Guide	Unwilling	1.82 (0.55–6.04)	82%	0.976	0.329
Guide	Body Sensitivity	1.45 (0.63–3.34)	45%	0.866	0.386
Hearing	Sex (M)	0.28 (0.08–0.92)	-72%	-2.105	0.035
Hearing	Fear of Strangers	0.45 (0.07–2.78)	-55%	-0.863	0.388
Hearing	Noise Fear	0.45 (0.18–1.11)	-55%	-1.741	0.082
Hearing	Excitable	2.82 (1.11–7.15)	182%	2.179	0.029
Hearing	Activated by Stress	1.21 (0.49–2.96)	21%	0.414	0.679
Hearing	Inhibited by Stress	2.34 (0.92–5.96)	134%	1.788	0.074
Hearing	Fear of Novel Objects	0.78 (0.33–1.83)	-22%	-0.569	0.569
Hearing	High Energy	0.54 (0.13–2.29)	-46%	-0.831	0.406

Outcome	Predictor	Odds Ratio (95% CI)	Percentage Change in Odds	z Value	p Value
Hearing	Fear of Elevated Areas	1.63 (0.55–4.86)	63%	0.873	0.382
Hearing	Social Manners	1.20 (0.51–2.83)	20%	0.422	0.673
Hearing	Poor Self Modulation	1.82 (0.51–6.48)	82%	0.925	0.355
Hearing	Barks Excessively	1.32 (0.40–4.38)	32%	0.453	0.65
Hearing	Yields Space	1.32 (0.17–10.33)	32%	0.267	0.79
Hearing	Movement Excites	0.32 (0.14–0.73)	-68%	-2.697	0.007
Hearing	Handler-Dog Team	0.70 (0.32–1.50)	-30%	-0.925	0.355
Hearing	Unwilling	1.04 (0.32–3.34)	4%	0.062	0.951
Hearing	Body Sensitivity	0.89 (0.42–1.86)	-11%	-0.322	0.747
Medical Alert	Sex (M)	0.78 (0.15–3.93)	-22%	-0.301	0.763
Medical Alert	Fear of Strangers	0.00 (0.00–330972453233587072.00)	-100%	-0.264	0.792
Medical Alert	Noise Fear	0.56 (0.16–1.95)	-44%	-0.917	0.359

Outcome	Predictor	Odds Ratio (95% CI)	Percentage Change in Odds	z Value	p Value
Medical Alert	Excitable	12.78 (2.40–67.97)	1178%	2.989	0.003
Medical Alert	Activated by Stress	0.12 (0.02–0.83)	-88%	-2.148	0.032
Medical Alert	Inhibited by Stress	2.22 (0.46–10.60)	122%	0.998	0.318
Medical Alert	Fear of Novel Objects	0.85 (0.24–3.04)	-15%	-0.247	0.805
Medical Alert	High Energy	2.55 (0.09–72.46)	155%	0.548	0.584
Medical Alert	Fear of Elevated Areas	0.00 (0.00–476398615458601444044886 00.00)	-100%	-0.236	0.813
Medical Alert	Social Manners	0.51 (0.17–1.52)	-49%	-1.199	0.231
Medical Alert	Poor Self Modulation	22.13 (2.12–231.26)	2113%	2.587	0.01
Medical Alert	Barks Excessively	0.84 (0.14–5.10)	-16%	-0.191	0.848
Medical Alert	Yields Space	0.00 (0.00–2381584105463729.50)	-100%	-0.264	0.792
Medical Alert	Movement Excites	0.74 (0.27–2.00)	-26%	-0.596	0.551

Outcome	Predictor	Odds Ratio (95% CI)	Percentage Change in Odds	z Value	p Value
Medical Alert	Handler-Dog Team	2.10 (0.84–5.23)	110%	1.591	0.112
Medical Alert	Unwilling	3.45 (0.72–16.50)	245%	1.55	0.121
Medical Alert	Body Sensitivity	0.80 (0.24–2.73)	-20%	-0.35	0.726
Mobility	Sex (M)	1.57 (0.69–3.57)	57%	1.08	0.28
Mobility	Fear of Strangers	1.22 (0.43–3.44)	22%	0.372	0.71
Mobility	Noise Fear	0.66 (0.35–1.27)	-34%	-1.246	0.213
Mobility	Excitable	1.23 (0.59–2.55)	23%	0.555	0.579
Mobility	Activated by Stress	1.44 (0.74–2.82)	44%	1.064	0.287
Mobility	Inhibited by Stress	1.59 (0.79–3.20)	59%	1.307	0.191
Mobility	Fear of Novel Objects	1.04 (0.56–1.92)	4%	0.122	0.903
Mobility	High Energy	0.72 (0.22–2.38)	-28%	-0.531	0.595
Mobility	Fear of Elevated Areas	1.45 (0.59–3.54)	45%	0.813	0.416

Supplementary Material

Outcome	Predictor	Odds Ratio (95% CI)	Percentage Change in Odds	z Value	p Value
Mobility	Social Manners	1.33 (0.70–2.53)	33%	0.867	0.386
Mobility	Poor Self Modulation	1.20 (0.48–3.02)	20%	0.385	0.7
Mobility	Barks Excessively	1.77 (0.65–4.81)	77%	1.118	0.264
Mobility	Yields Space	1.01 (0.19–5.37)	1%	0.009	0.993
Mobility	Movement Excites	0.72 (0.41–1.28)	-28%	-1.119	0.263
Mobility	Handler-Dog Team	1.47 (0.86–2.53)	47%	1.397	0.162
Mobility	Unwilling	1.94 (0.75–5.03)	94%	1.365	0.172
Mobility	Body Sensitivity	0.65 (0.36–1.18)	-35%	-1.423	0.155
Seizure Response	Sex (M)	1.08 (0.18–6.35)	8%	0.083	0.934
Seizure Response	Fear of Strangers	0.29 (0.02–3.47)	-71%	-0.975	0.329
Seizure Response	Noise Fear	0.95 (0.25–3.54)	-5%	-0.08	0.937

Outcome	Predictor	Odds Ratio (95% CI)	Percentage Change in Odds	z Value	p Value
Seizure Response	Excitable	3.59 (0.81–15.92)	259%	1.682	0.093
Seizure Response	Activated by Stress	1.40 (0.36–5.50)	40%	0.486	0.627
Seizure Response	Inhibited by Stress	1.42 (0.34–5.96)	42%	0.485	0.628
Seizure Response	Fear of Novel Objects	5.44 (1.39–21.32)	444%	2.431	0.015
Seizure Response	High Energy	5.01 (0.30–83.27)	401%	1.125	0.261
Seizure Response	Fear of Elevated Areas	3.89 (0.68–22.45)	289%	1.521	0.128
Seizure Response	Social Manners	1.06 (0.28–4.06)	6%	0.092	0.927
Seizure Response	Poor Self Modulation	2.33 (0.24–22.25)	133%	0.735	0.462
Seizure Response	Barks Excessively	0.41 (0.04–3.95)	-59%	-0.769	0.442
Seizure Response	Yields Space	4.64 (0.32–66.70)	364%	1.129	0.259

Supplementary Material

Outcome	Predictor	Odds Ratio (95% CI)	Percentage Change in Odds	z Value	p Value
Seizure Response	Movement Excites	0.64 (0.20–2.12)	-36%	-0.722	0.47
Seizure Response	Handler-Dog Team	1.03 (0.36–2.96)	3%	0.051	0.959
Seizure Response	Unwilling	1.06 (0.19–6.01)	6%	0.062	0.95
Seizure Response	Body Sensitivity	0.12 (0.02–0.68)	-88%	-2.387	0.017
Veteran/PTSD	Sex (M)	3.67 (1.27–10.63)	267%	2.399	0.016
Veteran/PTSD	Fear of Strangers	0.54 (0.19–1.57)	-46%	-1.132	0.258
Veteran/PTSD	Noise Fear	0.73 (0.35–1.50)	-27%	-0.86	0.39
Veteran/PTSD	Excitable	1.05 (0.43–2.56)	5%	0.098	0.922
Veteran/PTSD	Activated by Stress	1.70 (0.74–3.87)	70%	1.258	0.208
Veteran/PTSD	Inhibited by Stress	2.32 (0.89–6.01)	132%	1.728	0.084
Veteran/PTSD	Fear of Novel Objects	1.45 (0.69–3.07)	45%	0.972	0.331
Veteran/PTSD	High Energy	1.33 (0.41–4.27)	33%	0.479	0.632

Outcome	Predictor	Odds Ratio (95% CI)	Percentage Change in Odds	z Value	p Value
Veteran/PTSD	Fear of Elevated Areas	0.74 (0.29–1.87)	-26%	-0.633	0.526
Veteran/PTSD	Social Manners	1.55 (0.72–3.37)	55%	1.117	0.264
Veteran/PTSD	Poor Self Modulation	0.69 (0.26–1.83)	-31%	-0.743	0.457
Veteran/PTSD	Barks Excessively	1.39 (0.41–4.68)	39%	0.525	0.599
Veteran/PTSD	Yields Space	1.84 (0.33–10.40)	84%	0.692	0.489
Veteran/PTSD	Movement Excites	0.53 (0.25–1.10)	-47%	-1.716	0.086
Veteran/PTSD	Handler-Dog Team	1.10 (0.51–2.38)	10%	0.244	0.808
Veteran/PTSD	Unwilling	0.99 (0.30–3.31)	-1%	-0.014	0.989
Veteran/PTSD	Body Sensitivity	0.85 (0.43–1.67)	-15%	-0.478	0.633
Autism	Sex (M)	0.34 (0.01–9.68)	-66%	-0.63	0.529
Autism	Fear of Strangers	0.00	-100%	-0.177	0.859

Supplementary Material

**Outcome**	**Predictor**	**Odds Ratio (95% CI)**	**Percentage Change in Odds**	**z Value**	**p Value**
		(0.00–11120658114177088440686 8248648268848064426.00)			
Autism	Noise Fear	7.07 (0.58–85.48)	607%	1.538	0.124
Autism	Excitable	0.01 (0.00–0.16)	-99%	-3.163	0.002
Autism	Activated by Stress	0.58 (0.03–11.77)	-42%	-0.355	0.723
Autism	Inhibited by Stress	0.02 (0.00–0.28)	-98%	1.728	0.084
Autism	Fear of Novel Objects	0.32 (0.03–3.97)	-68%	-0.89	0.373
Autism	High Energy	0.11 (0.00–18.76)	-89%	-0.848	0.397
Autism	Fear of Elevated Areas	1125.78 (0.00–18909932692142821116284 4286440244.00)	112478%	0.205	0.838
Autism	Social Manners	0.34 (0.03-4.00)	-66%	-0.855	0.393

**Outcome**	**Predictor**	**Odds Ratio (95% CI)**	**Percentage Change in Odds**	**z Value**	**p Value**
Autism	Poor Self Modulation	0.01 (0.00-0.31)	-99%	-2.545	0.011
Autism	Barks Excessively	11.37 (0.14-932.41)	1037%	1.081	0.28
Autism	Yields Space	213.36 (0.00–174259957658720894986.00)	21236%	0.255	0.799
Autism	Movement Excites	87.63 (9.20-834.91)	8663%	3.889	<0.001
Autism	Handler-Dog Team	0.27 (0.03-2.35)	-73%	-1.183	0.237
Autism	Unwilling	0.04 (0.00-1.34)	-96%	-1.794	0.073
Autism	Body Sensitivity	10.53 (0.75-148.54)	953%	1.744	0.081

6 Distributions among Dogs Released from Training

For behavioral releases, the largest proportion of dogs were released because of environmental soundness (12.5%, n=112). The rest of the categories of behavioral release were relatively evenly distributed between 1-10% of the behavioral release subpopulation. For medical releases, the overwhelming majority of dogs were released for skin allergies (suspected or diagnosed, 37%, n=108). The remaining categories were all 10% or less of the medical release subpopulation.

Supplemental Table 7. Release reasons for behavioral release and medical release.

Release Type	Release	Number of Dogs	Percentage
Behavior	Environmental Soundness	112	12.5%
	Inhibited by Stress	79	9%
	Arousal (excitable)	76	8.5%
	Stranger Fear/Aggression	72	8%
	Activated by Stress	67	7.5%
	Dog Fear/Aggression	60	7%
	Adaptability	55	6%
	Resilience	51	6%
	Body Sensitivity	48	5%
	Excessive Barking/Whining	38	4%
	Initiative/Drive	29	3%
	Anxious in New Locations	28	3%
	Resource Guarding	16	2%
	Manners with People	15	2%
	Inappropriate Elimination	11	1%
	Chasing/Movement Excites	10	1%

Release Type	Release	Number of Dogs	Percentage
	Riding/Vehicle Issues	10	1%
	Relationship Skills	7	<1%
	Scavenges	7	<1%
	Manners (Destructive)	5	<1%
	Elevation Fear	4	<1%
	Underfooting Fear	4	<1%
	Manners (Scavenges)	3	<1%
	Aggression when Challenged	2	<1%
	Thunderstorm Fear	2	<1%
	Stress Lack of Focus	1	<1%
	Total	*812*	*95.5% (remaining 4.5% removed for unspecified behavior release)*
Medical	Skin Allergies (suspected or diagnosed)	108	37%
	Ophthalmologic Eyes	29	10%
	Digestive	23	8%
	Hips	20	7%
	Elbows	17	6%
	Seizures/Tremors	13	4%
	Cardiac	11	4%
	Urogenital	10	3.5%
	Musculoskeletal	9	3%
	Otitis Ear Infection	8	3%
	Conformation/Gait	5	2%

Supplementary Material

**Release Type**	**Release**	**Number of Dogs**	**Percentage**
	Cancer	4	1%
	Neurologic (not Seizure/Tremor)	4	1%
	Congenital Deformity	3	1%
	Accident, Dog is Unusable	2	1%
	Autoimmune	1	<1%
	Dental	1	<1%
	Endocrine	1	<1%
	Respiratory	1	<1%
	Total	*270*	*95.5% (remaining 4.5% removed for unspecified medical release)*
